# Results from the TEHDAS country visits: State-of-play of 12 EU member states in relation to the EHDS2

**DOI:** 10.1093/eurpub/ckac129.366

**Published:** 2022-10-25

**Authors:** L Abboud, S Cosgrove, I Kesisoglou, P Bogaert

**Affiliations:** EU Health Information Systems, Sciensano, Brussels, Belgium

## Abstract

Successfully launching a sustainable EHDS requires a clear understanding of the current situation in Member States, taking into account the existing diversity. With this in mind, country visits are being carried out in a group of 12 countries: Denmark, Belgium, Hungary, Netherlands, Estonia, Ireland, Portugal, Finland, Czech Republic, Slovenia, Sweden, Germany. The aim of the country visits is to map the state-of-play of health data management in different countries and explore their preparedness to join the EHDS. The topics explored include: the health data sources available, the health data infrastructure (storage, access and interoperability), data quality assurance mechanisms, data governance, as well as resource and training needs and opportunities. Another aim of the country visits is to explore needs and expectations at national level, and how the EHDS can respond to them. This presentation will include an overview of the health data management systems of the 12 countries mapped, providing a snapshot of the situation across Europe. It will then present the findings on their preparedness to join the EHDS. Preparedness takes into account a variety of factors, including political will, as well as legal and technical factors. The presentation will further share the lessons learnt and best practices highlighted from the country visits. Finally, based on these findings, the presentation will close with an overarching view on the preparedness across Europe to deploy the EHDS for the secondary use of health data, EHDS2, as well as planned actions that would facilitate the implementation of the EHDS2 in different member states.

